# l-Alanine capping of ZnO nanorods: increased carrier concentration in ZnO/CuI heterojunction diode†

**DOI:** 10.1039/c7ra12385j

**Published:** 2018-01-31

**Authors:** E. Indubala, M. Dhanasekar, V. Sudha, E. J. Padma Malar, P. Divya, Jositta Sherine, Revathy Rajagopal, S. Venkataprasad Bhat, S. Harinipriya

**Affiliations:** Electrochemical Systems Lab, SRM Research Institute, SRM Institute of Science and Technology Kattankulathur Chennai – 603203 India harinipriya.s@res.srmuniv.ac.in; SRM Research Institute, SRM Institute of Science and Technology Kattankulathur Chennai – 603203 India; Department of Chemistry, SRM Institute of Science and Technology Kattankulathur Chennai – 603203 India; National Centre for Ultrafast Processes, University of Madras Taramani Campus Chennai – 600 113 India; Department of Physics and Nanotechnology, SRM Institute of Science and Technology Kattankulathur Chennai – 603203 India; Department of Chemistry, Stella Maris College Chennai – 600086 India

## Abstract

ZnO nanorods were capped with a simple amino acid, *viz.*, l-alanine to increase the carrier concentration and improve the performance of ZnO/CuI heterojunction diodes. The effect of l-alanine capping on the morphology, structural, optical, electrochemical and electrical properties of ZnO nanorods had been studied in detail. The stable structure with two equally strong Zn–O coordinate bonds predicted by density functional theory was in agreement with the experimental results of FTIR spectroscopy. Due to the presence of electron-releasing (+I effect) moieties in l-Alanine, the carrier concentration of capped ZnO nanorods was two orders of magnitude higher and the ZnO/CuI heterojunction device showed more than a two-fold increase in the photovoltaic power conversion efficiency.

## Abstract 2

ZnO nanorods were capped with a simple amino acid, *viz.*, l-Alanine to increase the carrier concentration and improve the performance of ZnO/CuI heterojunction diodes.

## Graphical abstract

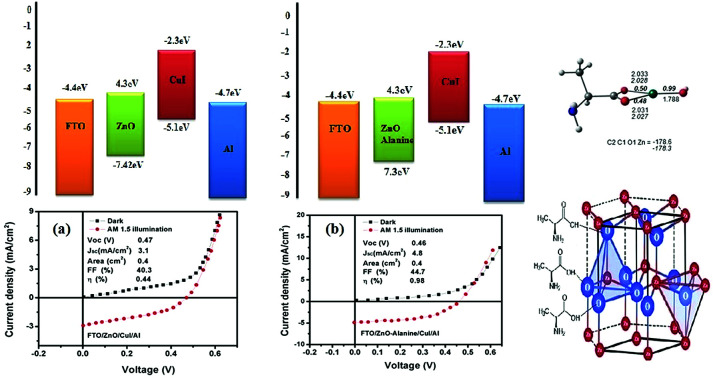

## 1 Introduction

Ligands are ions or molecules attached to the central metal atom *via* coordinating bonds. By attaching two or three different ligands to the surface of a single nanoparticle, electric fields large enough to significantly alter the electronic and optoelectronic properties of the nanocrystals could be induced. Ligands such as amino acids, polyamines, and thiols can act as good capping agents due to their inductive effect and surface induced dipoles on the capped materials. Surface capping of nanocrystals with mixed ligands plays a vital role to enhance the performance of quantum dots (QDs) based optoelectronic devices *via* tuning of the electronic energy levels. The energy level tuning depends strongly on the surface linking groups and the ratio of the different capping molecules attached to the surface of the QDs.^1^ Ligand exchange and complexation are widely used to modify the electronic properties of semiconductor QDs such as CdS,^2^ CdSe,^3–5^ and PbSe^4^*via* ligand induced surface dipoles.

Transparent conducting materials such as ZnO and Al-doped ZnO have been utilized as unipolar n-type semiconductors^6–9^ in diode applications.^10,11^ Capping, surface modification and substitution of ZnO for various applications have been studied extensively in the literature.^1,12–19^ Thus, a simple route to improve the carrier concentration of n-type semiconductors such as ZnO and to enhance the efficiency of heterojunction and photovoltaic devices has become inevitable. Surface modification of ZnO nanoparticles (NPs) by *n*-propylamine and thiols influences the electronic memory effect of ZnO–polystyrene diodes.^12^ Methods of surface modification and the influence of surface substitution on the electronic properties of ZnO surfaces at molecular interfaces of hybrid photovoltaic (hPV) and organic photovoltaic (OPV) devices have been extensively studied in the literature.^15^ Surface modified ZnO NPs showed significant enhancement in the spectrum selectivity and absorption efficiency in the UV range.^16^ The 1,2-ethanedithiol (EDT) doped ZnO composite behaved as an electron transporting layer to improve the performance of inverted polymer solar cells.^17^ The strong interaction between ZnO and the –SH group of EDTs contributed to the formation of a homogeneous film of ZnO–EDT by passivating the surface defects and increasing the electron mobility of ZnO effectively. It was demonstrated in the literature that ZnO–EDT increased the electron extraction and facilitated more photoinduced carrier generation than unpassivated ZnO. The space-charge limited current (SCLC) measurements were performed to determine the change in electron mobility of ZnO with and without doping.^20,21^ The electron mobility of the device with ZnO–0.5% EDT was more than double of that with bare ZnO; it was suggested that the higher electron mobility may be due to the good crystallinity and surface defect passivation.^17^ According to another report,^22^ band gap engineering of PbS QDs *via* encapsulation of PbS using complexing ligands such as EDT increased the efficiency of the ZnO/PbS heterojunction upto 8.55%.

Surface modification of ZnO NPs by amino acids such as l-Alanine and l-Arginine has been reported in the literature^22,23^ for band gap engineering and thermo-optic applications.^23^l-Arginine capped Nd^3+^ and Li^+^ co-doped CdS QDs have been utilized in the literature^24^ to demonstrate a large blue shift in band gap energy, as compared to that of bulk CdS, due to the quantum confinement effect. Water soluble CuS QDs have been prepared efficiently using alanine as a bio-compatible organic capping molecule.^25^ In the present study, we employed a simple precipitation method to synthesize ZnO nanorods (NRs) with l-Alanine as the capping agent to improve the carrier concentration and performance of ZnO/CuI and ZnO–l-Alanine/CuI heterojunction diodes, respectively. Although several amino acids can be used for the present study, alanine was chosen as it is the simplest chiral amino acid available abundantly in nature. Specifically, the l-enantiomer of alanine was preferred to accommodate effective coordination bonding with ZnO NRs. In addition, the electron-releasing (+I effect) of –CH_3_, –COOH and –NH_2_ groups present in alanine facilitated the electron-rich surface for ZnO by passivating the surface defects and increasing the electron mobility of ZnO effectively. Due to the high aspect ratio, one-dimensional electronic percolation and carrier mobility in NRs enabled it to be a feasible candidate for capping studies in comparison with other geometries.^26^ Studies on the effect of l-Alanine capping on the structural, optical, electrical and electrochemical properties of ZnO NRs were carried out in detail. Theoretical investigations based on density functional theory were performed to understand the structural stability and geometry of ZnO–l-Alanine that supports the carrier concentration increase due to the +I effect. Further, the positive contribution of this +I effect on the photocurrent generation in the ZnO/CuI heterojunction device was also demonstrated.

## 2 Experimental section

### 2.1 Synthesis of ZnO NRs

Zinc sulfate (1.5 mol L^−1^) and ammonium bicarbonate (2.5 mol L^−1^) were prepared in distilled water and 100 mL of ZnSO_4_ solution was added to 126 mL of NH_4_HCO_3_ solution with stirring at 45 °C. The slurry of basic zinc carbonate (BZC) in the form of a white precipitate was obtained. It was then filtered, washed and dried. The precipitate was calcined at 500 °C for 1 h to obtain ZnO NRs.

### 2.2 Synthesis of l-Alanine capped ZnO NRs

Initially, 0.2 g of ZnO NRs was diluted with deionized water and stirred for 2 h using a magnetic stirrer. After 2 h, 2 g of l-Alanine, obtained commercially from Sigma Aldrich, India, was added into the ZnO solution and then, the mixture was stirred for up to 24 h. After stirring, ZnO–l-alanine composites were separated by centrifugation at 1000 rpm for 15 min. The separated precipitate was dried in a hot-air oven for 24 h to obtain ZnO–l-Alanine.

### 2.3 Synthesis of CuI nanoflowers from *Syzygium cumini* extract

*Syzygium cumini* or naval fruit seeds were washed several times thoroughly under running water and rinsed with distilled water. The cleaned seeds were then soaked in distilled water for 24 h. The soaked seeds were ground into a paste using a mixer. The resultant paste was diluted with distilled water and stirred continuously for 3 h to attain homogeneity. The subsequent solution was then centrifuged. The centrifugate was used for synthesis of CuI nanoflowers. Cu(NO_3_)_2_·3H_2_O and NaI were purchased from Qualigens and SDFCL, respectively, and were used without further purification. Initially, 30 mL of *Syzygium cumini* extract was added dropwise into 2 g of Cu(NO_3_)_2_·3H_2_O, which was dissolved in 75 mL of distilled water under magnetic stirring. Then, 1.2408 g of NaI dissolved in 75 mL of distilled water was added dropwise into the above solution. The obtained mixture was stirred at room temperature. The resultant dark brown precipitate was filtered, washed thoroughly with 50% ethanol and dried at 50 °C.

### 2.4 Materials characterization

XRD of ZnO, ZnO–l-Alanine and CuI were obtained using a Bruker D8 Advance diffractometer using CuKα radiation and *λ* = 1.5406 Å. The morphology of the above-mentioned compounds was analyzed by SEM-EDS analysis (Carl Zeiss-SEM, EDS-Oxford Instruments). Surface morphology was studied by a metallurgical microscope (LEICA DM Model no. 1). Optical measurements were carried out using a UV-Vis spectrophotometer (Specord 200 plus, Analytikjena, Germany) in diffuse reflectance spectral (DRS) mode. FTIR of ZnO and ZnO–l-Alanine NRs were performed by Bruker FTIR. Photoluminescence spectroscopic analysis of ZnO and l-alanine capped ZnO NRs was carried out using a Fluorolog Horiba spectrophotometer. The surface morphology and structural studies of green-synthesized CuI nanoflowers are provided in Fig. S1 and S2 and Table S1 of the ESI.†

### 2.5 Quantum chemical study

The interaction between l-Alanine and ZnO molecule was studied in the gas-phase starting from the lowest energy alanine conformer^27^ (Ala-I, *cf.* Section 3.5). Different probable modes of capping of ZnO with the Ala-I conformer leading to complex formation were examined. Structures of the Ala–ZnO complex, in which the carboxylic hydrogen of alanine adopts a *trans* arrangement, and Ala-Ia were also investigated. The complex geometries were subjected to complete structural optimization using meta-hybrid density functional theory (DFT) method M05-2X^28^ and double-hybrid DFT method B2PLYP,^29^ which were found to be suitable to study covalent as well as noncovalent interactions. Aldrich's triple-zeta valence polarized basis set TZVP^30^ in the M05-2X calculation and the extended triple-zeta valence basis set def2-TZVP,^31^ including the auxiliary def2-TZVP/J^32^ basis set in the B2PLYP, was used in this study. The above basis sets were reported to yield reliable results in quantum chemical studies.^33^ In order to accurately predict the relative energies of the alanine–ZnO structures, single-point coupled-cluster calculations with single, double and perturbative triple excitations CCSD(T)^34,35^ were performed. This method was considered to be the “Gold Standard” method in quantum chemistry. CCSD(T) calculations were carried out on the M05-2X/TZVP and B2PLYP/def2-TZVP optimized geometries using polarized valence triple-zeta basis set TZVP.^31^ Domain based local pair natural orbital CCSD(T) [DLPNO-CCSD(T)]^36^ calculations using the extended def2-TZVP^31^ basis set, including auxiliary def2-TZVP/J^33^ and def2-TZVP/C,^37^ were also performed for the alanine–ZnO complex structures. The suitability of the DLPNO-CCSD(T)/def2-TZVP method to predict the relative energies was tested by performing rigorous CCSD(T)/QZVP calculations on the M05-2X/TZVP optimized structures A and B of the alanine–ZnO complex. The M05-2X calculations were performed using the Gaussian 03 software.^38^ B2PLYP and coupled cluster calculations were carried out by the ORCA 4.0.0 software.^39^ In the B2PLYP and the DLPNO-CCSD(T) calculations, the combination of the resolution of the identity (RI)^32^ and the “chain of spheres exchange” algorithms (RIJCOSX)^40–44^ was implemented in the ORCA software.

### 2.6 Electrochemical characterization

Cyclic voltammetry studies were performed using a typical three-electrode system. ZnO and ZnO–l-Alanine NRs were coated by drop casting on a glassy carbon electrode that served as the working electrode, a platinum wire was used as the counter electrode and Ag/AgCl was used as the reference electrode. The electrolyte used was 0.1 M KCl. Electrochemical impedance spectroscopy of the FTO/ZnO/CuI/Al and FTO/ZnO–l-Alanine/CuI/Al heterojunction devices were carried out using an Ivium pocketstat electrochemical workstation in two-electrode solid-phase mode.

### 2.7 Heterojunction device fabrication

The heterojunction devices were fabricated with the structure FTO/ZnO/CuI/Al. Fluorine-doped tin oxide (FTO) coated glass substrate (Sigma Aldrich, TEC-7, 2 mm thickness) was cleaned with DI water and then with ethanol for 10 min in an ultrasonic bath, dried in air and heated at 250 °C for 5 min to remove any residual organics. ZnO or ZnO–l-Alanine NRs dispersed in ethanol were coated on the substrate by the doctor blade method. CuI dissolved in acetonitrile was spin coated on the FTO/ZnO or FTO/ZnO–l-Alanine films. Adhesive Al conductive foil was stacked on top of the films for electrical contact and the active area of the heterojunction device was ∼0.4 cm^2^ (illustrative representation and photograph of the devices are provided in ESI, Fig. S3†). The devices were tested using a Keithley 2450 (Tektronix, USA) source measure unit and a solar simulator (Science Tech, 150 W, Class ABA), providing AM 1.5 illumination at 100 mW cm^−2^ light intensity.

## 3 Results and discussion

The results showed that the capping of ZnO NRs by l-Alanine occurred *via* coordination bonding between zinc of ZnO and oxygen of the carboxylic group of the amino acid. Through this coordination bonding, the electron flow from l-Alanine molecules to the surface of ZnO occurred and resulted in increased carrier concentration of ZnO NRs as demonstrated in the following sections.

### 3.1 Structural analysis

**Fig. 1:**
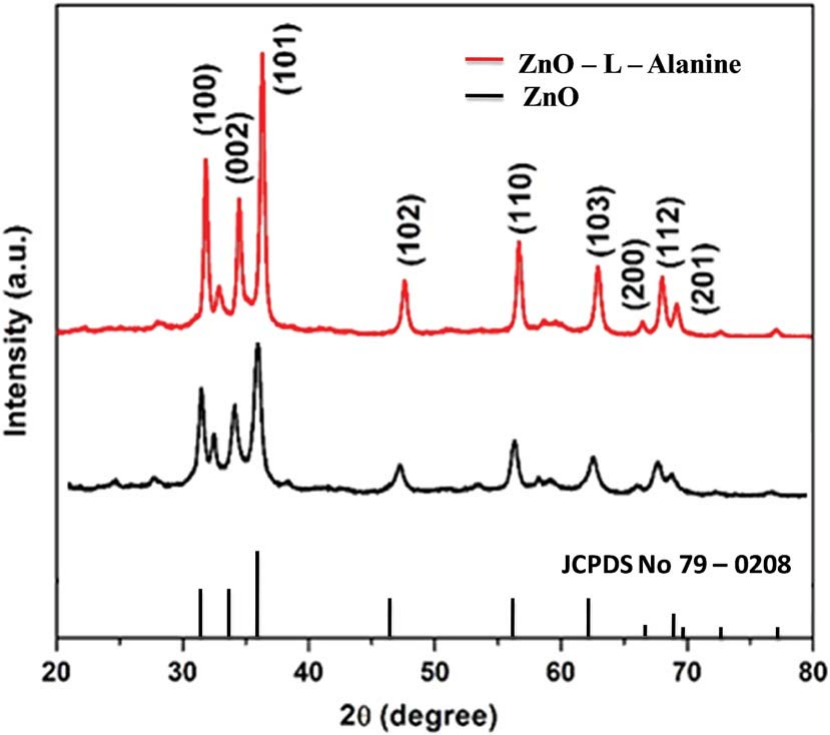
XRD patterns of ZnO and ZnO–l-Alanine NRs.

The powder X-ray diffraction patterns of ZnO and ZnO–l-Alanine NRs were provided in Fig. 1. The crystallite size of ZnO and ZnO–l-Alanine NRs was calculated as 13–16 nm using the Scherrer equation.^45^ All diffraction peaks could be indexed to the hexagonal crystal structure of ZnO with cell parameters of *a* = 3.264 Å and *c* = 5.219 Å (JCPDS file no. 79-0208). The 2*θ* values of 31.61°, 34.33°, 36.10°, 47.36°, 56.31°, 62.64°, 66.03°, 67.64°, and 68.73° could be attributed to the crystal planes (100), (002), (101), (102), (110), (103), (200), (112) and (201), respectively. Peak broadening^25^ could be observed in case of the ZnO NRs prepared using capping agents, which confirmed the coordination bonding between ZnO and C=O of alanine, resulting in agglomeration (*cf.* Section 3.5). XRD patterns also confirmed the high purity of the nanorods as no peaks related to impurities were observed.

### 3.2 Morphology, composition and dispersion

**Fig. 2:**
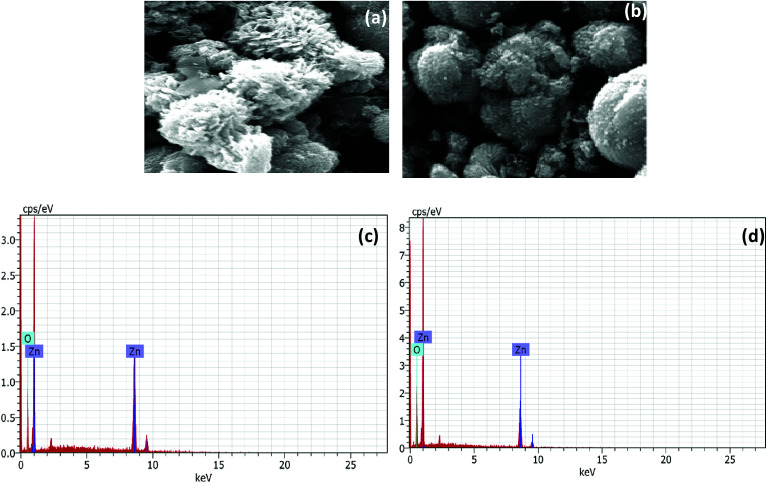
SEM of (a) ZnO and (b) ZnO–l-Alanine NRs; EDS of (c) ZnO NRs and (d) ZnO–l-Alanine NRs.

**Fig. 3:**
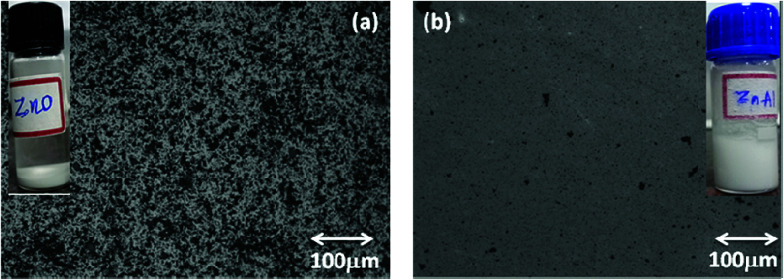
Optical microscopy images of (a) ZnO and (b) ZnO–l-Alanine NRs film coated on a glass substrate. Insets in (a) and (b) show the dispersion of the NRs in ethanol used for doctor blade coating, which were kept for six months.

The SEM analysis revealed the morphology of agglomerated ZnO NRs having a diameter of 200 nm (Fig. 2a). The l-Alanine capped NRs were more agglomerated in the form of microspheres (Fig. 2b). The EDS pattern (Fig. 2c and d) of the as-synthesized ZnO NRs and l-Alanine capped ZnO NRs confirmed the purity of the prepared samples. The EDS pattern showed peaks corresponding to Zn and O. Energy peaks corresponding to the elements C and N were due to the presence of l-Alanine. The dispersion of l-Alanine capped ZnO NRs were stable upto 6 months, while ZnO NRs settled down in ethanol within 10 min as shown in the photographs of the insets of Fig. 3. The surface morphology of ZnO and ZnO–l-Alanine films were studied using optical microscopy. The optical microscopy image indicated the presence of uniform grains on the glass substrate for l-Alanine capped ZnO NRs in comparison with uncapped ZnO NRs (Fig. 3). The films were continuous, adhered well to the substrate and did not fall off. This allowed for smooth fabrication and testing of the heterojunction devices (the photographs of NR films as well as heterojunction films are included in Fig. S3 of ESI†).

### 3.3 Absorption and photoluminescence studies

**Fig. 4:**
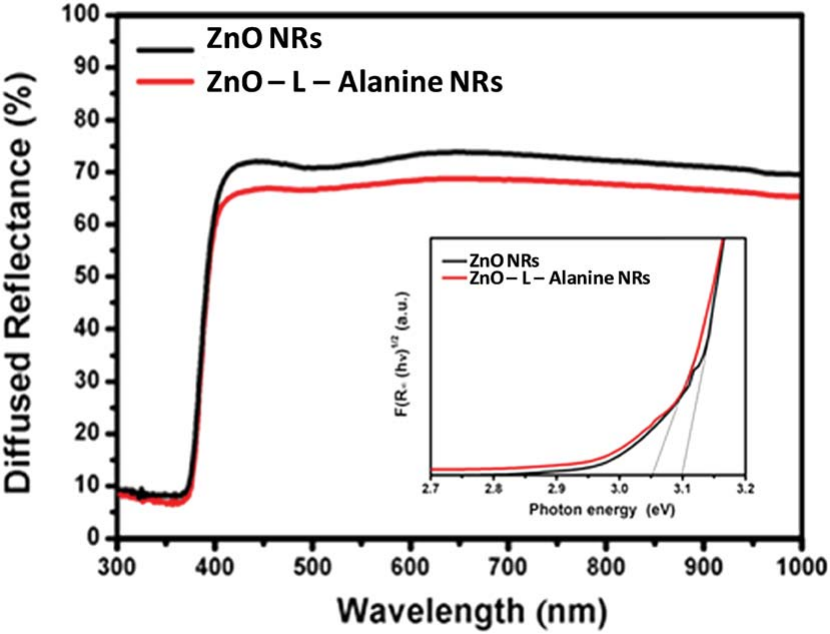
UV-diffuse reflectance spectra of ZnO and ZnO–l-Alanine NRs. Inset: estimation of bandgap of ZnO and ZnO–l-Alanine NRs.

The photoluminescence (PL) spectra of the aqueous suspension of ZnO were recorded on a Fluorolog-2500 fluorescence spectrophotometer. For PL measurement, the water suspensions of the samples were excited to the wavelength of 320 nm. DRS spectra of ZnO and ZnO–l-Alanine NRs are provided in Fig. 4. The graph of (*F*(*R*_*α*_)*hν*)^1/2^*versus hν* from Kubelka–Munk function^27^ (where *F*(*R*) is the K–M function, *R* represents the diffuse reflectance, and *hν* depicts the photon energy) was plotted as shown in the inset of Fig. 4. The band gap was obtained from the intercept of the linear part of the graph on the *x*-axis. ZnO NRs exhibited an absorption edge at ∼400 nm and the corresponding intrinsic band-gap energy was calculated as 3.12 eV, which was in agreement with the literature.^46^ The lower band gap for ZnO NRs in comparison with the band gap at 3.3 eV for bulk ZnO could be attributed to the agglomeration of the NRs in the form of nanospheres^46^ (*cf.*Fig. 2a). For ZnO–l-Alanine, the absorption peak shifted to the visible region and the band gap was estimated to be 3.05 eV. This lowering of the band gap could be justified by the increase in the carrier concentration of ZnO upon capping with l-Alanine. Due to the increased electron concentration in the valence band, the energy required to get excited to the conduction band decreases.^46^

**Fig. 5:**
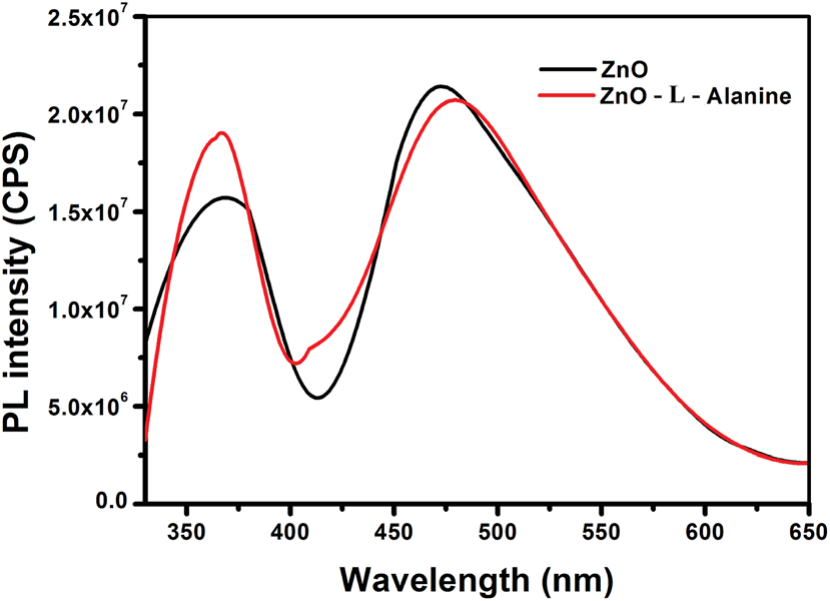
PL spectra of ZnO and ZnO–l-Alanine NRs.

In the PL spectra (Fig. 5), the ZnO NRs exhibited two characteristic emissions: one in the UV region around 370 nm and another in the visible region around 475 nm. The UV emission of ZnO NRs showed a blue shift of 20 nm in comparison to that of bulk ZnO. The blue shift was caused by the reduction in the particle size from bulk to nanostructure.^47,48^ The high intensity peak in the visible region around 475 nm was attributed to the oxygen vacancies on the surface of ZnO NRs.^47,48^l-Alanine capped ZnO NRs (red line in Fig. 5) showed a slight red shift in the UV band emission (at 375 nm) when compared to uncapped ZnO NRs. This slight red shift could be due to the aggregation of the particles during capping of the ZnO NRS by l-Alanine.^47,48^ In addition, we observed an increase in the intensity of the UV emission of ZnO–l-Alanine NRs as compared to that of ZnO NRs. The defect level emission for ZnO–l-alanine was observed at 485 nm with a 10 nm blue shift as compared to that of ZnO NRs. Information about the surface defects of the nanostructures could be obtained from the ratio of intensities of UV emissions (*I*_UV_) to that of defect level emissions (*I*_DL_). In the present scenario, (i) for ZnO NRs, *I*_UV_/*I*_DL_ was 0.7529 and (ii) for ZnO–l-Alanine NRs, *I*_UV_/*I*_DL_ was obtained as 0.9512. An increase in the intensity ratio with agglomeration of ZnO NRs upon capping with l-Alanine (*cf.*Fig. 2b) demonstrated a decrease in the visible emission caused by surface defects.^47,48^ Due to agglomeration, surface area of the ZnO NRs decreased (*cf.* Section 3.2), causing reduction in the surface area and defects. Thus, PL analysis indicated the surface passivation of ZnO NRs by capping with l-Alanine. These results are in conjunction with the frequency shift in FTIR, due to ZnO coordination of l-Alanine with C=O (Section 3.4), and the quantum chemical results (Section 3.5).

### 3.4 FTIR analysis

**Fig. 6:**
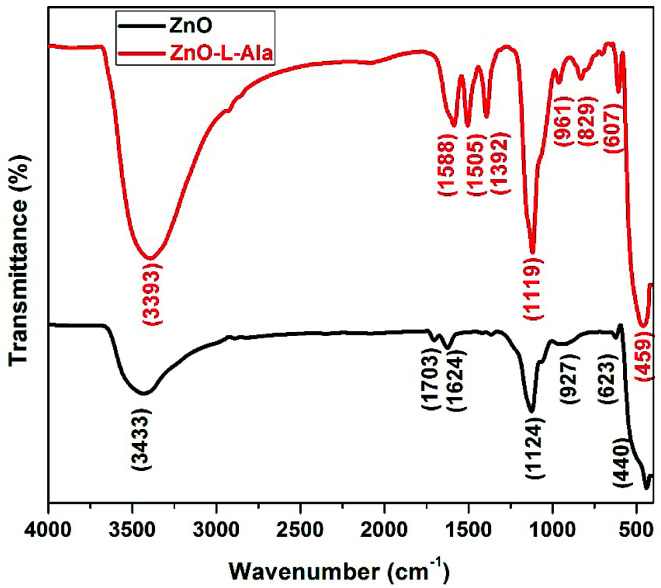
FTIR spectra of ZnO and ZnO–l-Alanine NRs.

The FTIR spectra of the ZnO and ZnO–l-Alanine NRs (Fig. 6) show a series of absorption peaks from 500 to 4000 cm^−1^. For ZnO NRs, peaks corresponding to a broad band at 3433 cm^−1^ were assigned to the O–H stretching mode of the hydroxyl group. The peaks observed at 1703, 1624, 1124, 927, and 623 cm^−1^ were due to the asymmetric and symmetric stretching of the zinc hydroxyl group. The peak at 440 cm^−1^ could be due to the stretching vibrations of ZnO.^23^ The hydroxyl groups on the surface of the ZnO NRs resulted from its hygroscopic nature.^23^ Collectively, these observations suggested that the FTIR-identified impurities primarily existed near the ZnO surface. In case of ZnO–l-Alanine, the FTIR spectrum showed a peak at 3398 cm^−1^ due to the O–H stretching. As the interaction between the –COOH group of l-Alanine with ZnO was through the coordination bonding of the type O–Zn–O=CHO–Zn, as depicted in quantum chemical studies (see Section 3.5), the broadening of the peak at 3398 cm^−1^ with high intensity was observed. Peaks at 1588 and 1505 cm^−1^ could be attributed to the presence of the –C=O functional group. The peak at 1392 cm^−1^ represented the vibrational modes of the –COO group. The peak at 1119 cm^−1^ indicated the presence of –C–O in the capped ZnO NRs. The peaks at 961 and 829 cm^−1^ corresponded to the C–H group vibrations. The peak at 607 cm^−1^ was due to the COO group, and that at 500 cm^−1^ was caused by the bending torsional modes of the CNH_2_ group in alanine. The shift in the frequency could be attributed to the effect of the stress and strain developed during the coordination bonding of the –COOH group with ZnO NRs. Thus, FTIR analysis confirmed the coordination bonding interaction as the mode of bonding between ZnO NRs and l-Alanine, as discussed in Section 3.5.

### 3.5 Quantum chemical study of ZnO–l-Alanine interaction

**Fig. 7:**
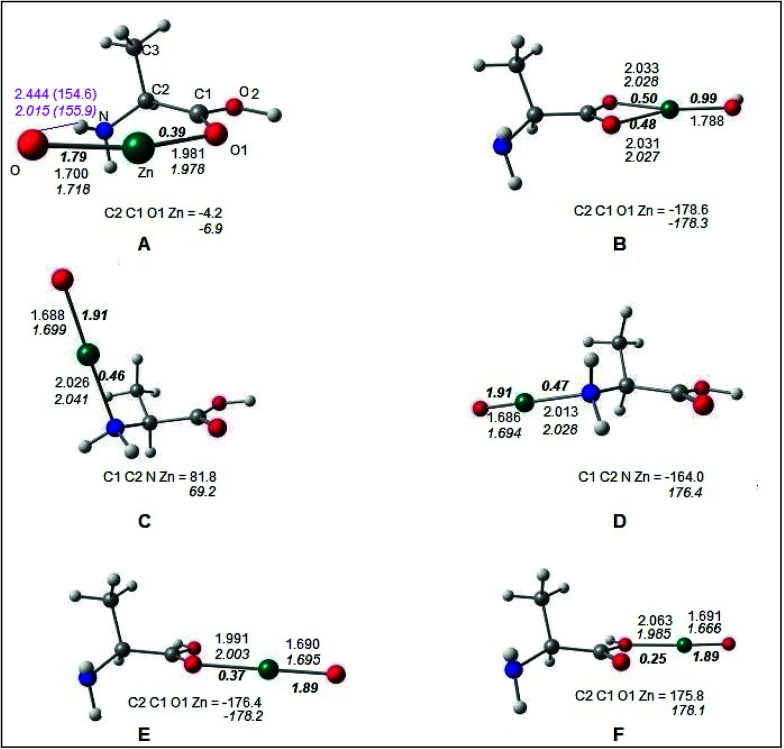
B2PLYP/def2-TZVP optimized geometries of l-alanine–ZnO complex. Labelling of atoms is shown in structure A. The ZnO⋯HN hydrogen bond in structure A is indicated with the purple line and the hydrogen bond length in Å and hydrogen bond angle in ° (inside parenthesis) are given in purple color. Bond lengths involving the Zn atom in Å and dihedral angle in ° are shown. The values in bold italics are covalent bond orders. Structural parameters in italics correspond to the optimized values obtained by the M052X/TZVP method. Color code for atoms: O – red; Zn – green; C – grey; H – white.

The optimized geometries of the Alanine–ZnO complex are shown in Fig. 7. It was found that the complex structures predicted by both M05-2X and B2PLYP methods were similar. All these structures corresponded to energy minima in the potential energy surface as proved by all the positive vibrational frequencies at the M05-2X/TZVP level. The complex structures A, B, C and D were characterized from the lowest energy Ala-I conformer. Structures E and F were formed from the alanine conformer with the carboxylic acid hydrogen in the *trans* form (Ala-Ia). The bonding between alanine and the ZnO molecules was analyzed by examining the covalent bond orders^49–51^ computed at B2PLYP/Def2-TZVP optimized geometries. In the structures A and B, ZnO was coplanar with the carboxylic acid group of alanine. In A, Zn formed coordinate covalent bond Zn–O1 with the carbonyl oxygen O1 of alanine, having a covalent bond order of 0.39 (Fig. 7). Oxygen of ZnO formed a hydrogen bond with the amino hydrogen (ZnO⋯HN). The hydrogen bond is indicated by the purple line in Fig. 7. The hydrogen bond length and angle were predicted to be 2.444 Å and 154.6° (2.015 Å and 155.9 Å), respectively, according to B2PLYP (M05-2X) calculations. In the discussion that follows, M05-2X results are included within parenthesis. Attempts to form hydrogen bonds between the OH group of alanine and ZnO yielded the structure B, in which the Zn atom was coordinately bound to both carboxylic oxygen atoms, leading to covalent bond orders of 0.48 and 0.50 for the bonds Zn–O1 and Zn–O2, respectively. It was remarkable that in structure B, Ala-I conformer behaved like a bidentate ligand with ZnO and the Zn–O bond became a single bond having a covalent bond order of 0.99. The OH hydrogen of Ala migrated to the oxygen of ZnO, forming a covalent O–H bond. Structures C and D were formed by capping of ZnO through coordinate bond formation with amino nitrogen atom in Ala-I in a monodentate fashion. In structure C, ZnO approached Ala-I almost perpendicular to the plane containing the O1, O2, C1, C2 and N atoms with the dihedral angle C1C2NZn = 81.8° (69.2°). The approach of ZnO was along the plane of O1, O2, C1, C2 and N atoms in the structure D and was in the *trans* arrangement to the NC2 bond as reflected by the dihedral angle C1C2NZn = −164.0° (176.4°).

The monodentate coordination mode was predicted between ZnO and carboxylic oxygen atoms of Ala-Ia, leading to structures E and F. In these structures, the ZnO was coplanar to the carboxyl group of Ala-Ia. Attempts to characterize the bidentate coordination between Ala-Ia carboxylic oxygen atoms and ZnO (similar to that of structure B) were unsuccessful. It was observed that the bond in ZnO was essentially a double bond in structures C, D, E and F, having covalent bond orders of 1.91 in C and D and 1.89 in E and F. The Zn–O bond length in the above structure was ∼1.69 Å. In the hydrogen bonded structure A, the Zn–O bond was slightly longer at 1.70 Å (1.72 Å) and the Zn–O covalent bond order was lowered to 1.79. In the bidentate structure B, the Zn–O bond was elongated to 1.788 Å as it had become a single bond (bond order ∼ 1) due to the migration of the hydroxyl hydrogen to this oxygen center. It was observed that the Zn–O coordinate bond with carboxylic oxygen atom(s) was longer (1.98–2.06 Å) in the complex structures A, B, E and F and the corresponding covalent bond order was in the range of 0.25–0.50. The Zn–N coordinate bonds in C and D were 2.026 and 2.013 Å (2.041 and 2.028 Å), respectively, having covalent bond orders of 0.46 and 0.47, respectively. The covalent bond orders between Zn and O/N of alanine were in the range of 0.25 to 0.50 for different structures A, B, C, D, E and F, as predicted by the B2PLYP method. These values were in agreement with earlier studies on coordinate bonds between metal and ligand atoms obtained by B3LYP and BP86 DFT methods.^52–56^

**Fig. 8:**
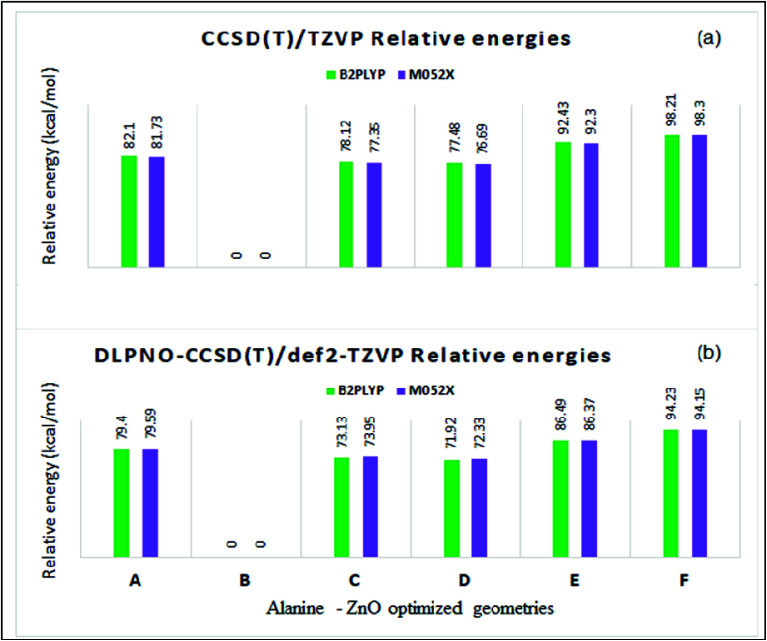
Relative energies (kcal mol^−1^) by (a) CCSD(T)/TZVP and (b) DLPNO-CCSD(T)/def2-TZVP methods in the ZnO–l-alanine optimized structures.

The total energies and relative energies of the optimized structures and the single-point CCSD(T) and DLPNO-CCSD(T) studies are shown in the ESI (Tables S2 and S3†). The total energies at different levels were corrected by adding zero-point vibrational energy (ZPE) obtained from the M05-2X/TZVP calculation. Relative energies of the complex structures with reference to the lowest energy structure at different levels are compared in Table S2 and S3.† The relative energies yielded by the CCSD(T)/TZVP and DLPNO-CCSD(T)/def2-TZVP single-point calculations are shown in Fig. 8a and b, respectively. The same order of relative stability among the different structures was predicted by the different methods used in the present study. The relative energy predicted by the DLPNO-CCSD(T) method (Fig. 8b) was about 2 to 6 kcal mol^−1^ less than the corresponding value obtained by the CCSD(T) study (Fig. 8a). The structure B is the lowest energy structure at the different levels of calculations. The coupled cluster relative energies obtained from B2PLYP and M05-2X geometries of a given complex structure differ within a range of 1 kcal mol^−1^. The stability of the structures in the decreasing order was as follows:B ≫ D ∼ C > A > E > F

The structure D with one Zn–N coordinate bond was ∼77 (∼72) kcal mol^−1^ higher in energy than the lowest energy structure B as predicted by CCSD(T)/TZVP (DLPNO-CCSD(T)/def2-TZVP) calculations. The dramatic stability of structure B was explained by the presence of two equally strong Zn–O coordinate bonds (bond orders of 0.48 and 0.50). The hydrogen bonded structure A was ∼15 kcal mol^−1^ higher in energy than structures C and D though A was formed through one Zn–O coordinate bond and one ZnO⋯HN hydrogen bond of medium strength (hydrogen bond energy ∼ −3 kcal mol^−1^). This trend can be explained from the predicted covalent bond order of 0.39 for the coordinate bond between the Zn atom and the carbonyl oxygen of alanine in structure A, which is weaker than the coordinate bonds in B, C and D, having a covalent bond order of ∼0.5. Further the ZnO bond was also weakened in A, as revealed by the lowering of the bond order to 1.7. The coordinate covalent bonds in E and F were also weak as the bond orders were 0.35 and 0.25, respectively, and thus account for their higher energies. In order to understand the role of the noncovalent dispersion energy in the relative stability of the different alanine–ZnO structures, the dispersion energy was computed using Grimme's DFT-D3 method at the M06-2X/def2-TZVP//M05-2X/TZVP level^57–61^(*cf.* ESI Tables S4 and S5†). It was observed that the dispersion energy contribution was small, in the range of −0.25 to −0.28 kcal mol^−1^ (Table S3†), and it had no significant role in the relative stability of the system under study. The present study revealed that the alanine–ZnO complex existed in the structure B. The analysis of the relative stabilities led to the inference that the presence of an additional Zn–O coordinate bond confers a stability of 70 kcal mol^−1^ to structure B as compared to structures A, C, D, E and F. This bidentate coordination of amino acids with metal oxides was experimentally demonstrated by Lambert *et al.*,^62^ thus supporting the structural stability of structure B through quantum mechanical studies. The reliability of the DLPNO-CCSD(T)/def2-TZVP results was checked by performing the more authentic CCSD(T)/QZVP calculations for the hydrogen bonded structure A and the capped structure B at M052X/TZVP optimized geometries. The relative energy of ∼79.5 kcal mol^−1^ predicted by the DLPNO-CCSD(T)/def2-TZVP was found to be closer to the value of 76.9 kcal mol^−1^ predicted by the CCSD(T)/QZVP method.

### 3.6 Electrical properties

The electrical properties of the ZnO and l-Alanine capped ZnO NRs films were investigated with the help of Hall effect measurements by employing the van der Pauw method. Table 1 indicates the Hall parameters for films of ZnO and ZnO–l-Alanine NRs. The carrier concentration of ZnO and ZnO–l-Alanine NRs were measured to be −9.875 × 10^17^ cm^−3^ and −9.131 × 10^19^ cm^−3^, indicating two orders of magnitude increase in the carrier concentration for ZnO NRs due to l-Alanine capping. The significant increase in the ZnO carrier concentration upon capping by l-Alanine could be attributed to the electron-releasing effect of the amino and carboxyl functional groups of the amino acids.^63^ The positive inductive effect (+I effect) of l-alanine led to electron pumping in the system *via* the coordination bonds, thus increasing the concentration of electrons in the ZnO–l-Alanine film.

**Table 1:** Results from Hall measurement of the ZnO and ZnO–l-Alanine NRs thin films

S. no.	Sample name	Carrier concentration (*P*) (cm^−3^)	Mobility (*μ*_H_) cm^2^ V^−1^ s^−1^	Electrical resistivity (Ω cm^−1^)
1	ZnO NRs	−9.875 × 10^17^	6.997	3.58 × 10^−3^
2	ZnO–l-Alanine NRs	−9.131 × 10^19^	11.880	3.087 × 10^−3^

### 3.7 Electrochemical studies

#### 3.7.1 Cyclic voltammetry analysis (CV)

**Fig. 9:**
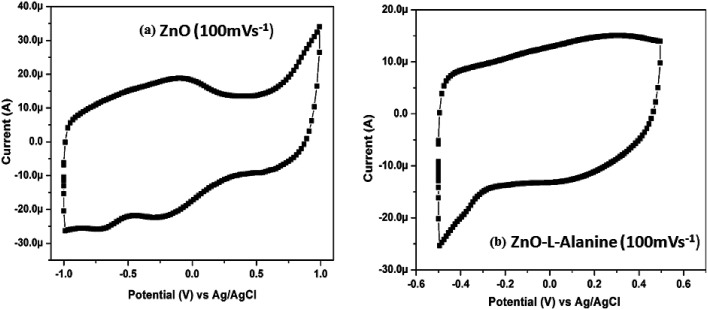
Cyclic voltammetry of the (a) ZnO and (b) ZnO–l-Alanine NRs at a scan rate of 100 mV s^−1^.

CV of ZnO NRs (Fig. 9) showed reduction peaks at −0.25 V and −0.7 V. The peaks corresponding to the reduction reactions (with respect to Ag/AgCl) as given below:ZnOH^+^ + H^+^ + e → Zn + H_2_O *E*^0^ = −0.741 VH^+^ + e → 1/2H_2_*E*^0^ = −0.244 V

CV of ZnO–l-Alanine NRs showed no redox peaks and behaved similar to that of a supercapacitor upon electrical perturbation. Thus, upon capping ZnO with l-Alanine, inhibition of reduction of ZnO into metallic Zn was observed. This behavior of ZnO–l-Alanine could be attributed to the +I effect of alanine functional groups. Capped ZnO became sufficiently electron rich and prevented further acceptance of electrons. Thus, ZnO–l-Alanine did not undergo any redox reaction upon electrical perturbation and behaved like a supercapacitor involving only mass and charge transfer. The capacitance of ZnO–l-Alanine was calculated from the CV curves as 1300 μF.

#### 3.7.2 Electrochemical impedance spectroscopy (EIS) analysis

**Fig. 10:**
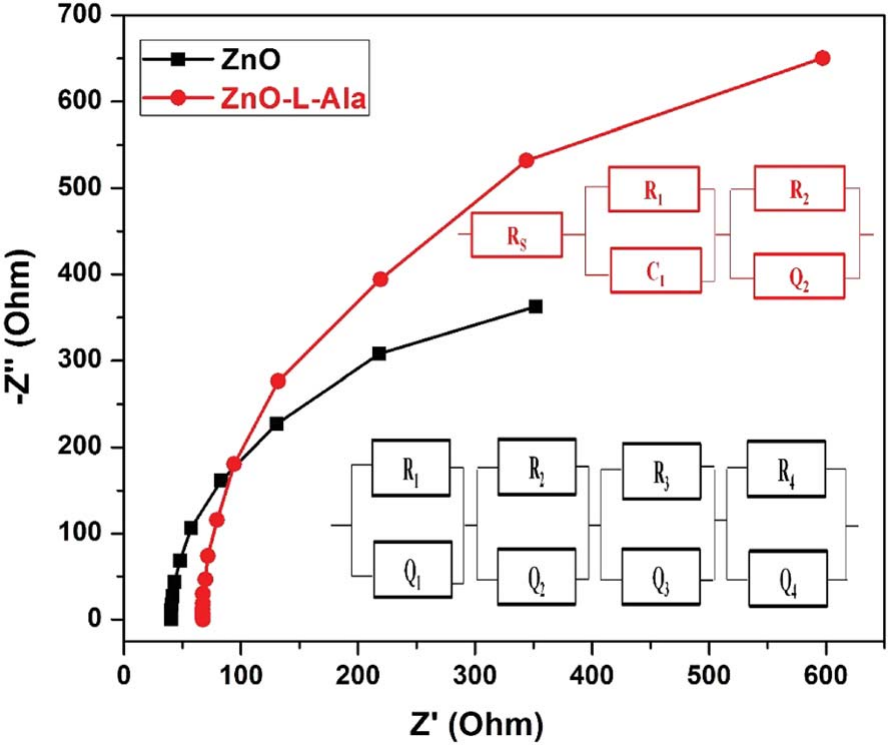
Nyquist plots for ZnO and ZnO–l-Alanine NRs along with their corresponding electrical equivalent circuit model used for data fitting.

Fig. 10 shows the Nyquist plot of ZnO/CuI (black line) and ZnO–l-Alanine/CuI (red line) heterojunction diode in the frequency range of 10 Hz to 100 kHz at an AC amplitude of 20 mV along with their corresponding electrical equivalent circuit model. *R*_1_*Q*_1_ circuit was fitted for the Nyquist plot of ZnO/CuI, where *R* represents the resistance and *Q* indicates the constant phase element (CPE). The surface inhomogeneity was high in the case of ZnO due to the agglomeration of the NRs as lumps on the substrate. The surface inhomogeneity and roughness associated resistance, *R*_1_, was obtained as 839.5 Ω, while *R*_2_, which is the resistance at ZnO/CuI interface, was obtained as 770.55 Ω. The void/CuI interfaces caused by surface inhomogeneity led to *R*_3_ with a value of 770.68 Ω. The CPE Qy1 corresponded to the capacitance at ZnO/CuI interface and was calculated as 490.97 mF (Qa1 value was near unity, *ca.* 0.9476). *C*_1_ demonstrated that the diffusion process dominated the heterojunction and the observed capacitance was due to charge accumulation at the ZnO/CuI interface. The second and third CPEs, Qy2 and Qy3, behaved as Warburg resistance, as their Qa components were very less (0.04 and 0.18, respectively). Thus, Qy2 and Qy3 values were obtained to be 0.015 Ω and 0.037 Ω, respectively. The incorporation of CPEs instead of capacitance in the equivalent circuit accounted more accurately for the heterogeneities including surface roughness, porosity and variation in the thickness^64^ of ZnO film coated by the doctor blade method. The *R*_1_*C*_1_*Q*_1_ circuit fitted nicely for the ZnO–l-alanine/CuI heterojunction device. The ohmic resistance (*R*_s_) was around 1.31 kΩ and the charge transfer resistance (*R*_1_) was an order of magnitude lower than the ohmic resistance, 67.07 Ω. The charge transfer capacitance *C*_1_ was very low, of the order of 51 μF. The Qy components behaved as pseudo capacitors with the exponent value of 0.99 and hence, they were taken to be *C*_2_ with a value of 490 μF. From the fitted values of *R*_s_, Qys and *C*_s_, it was clear that the *R*_1_ values of l-Alanine capped ZnO NR based devices were very less in comparison to those of uncapped ZnO NRs. The number of CPEs accounting for the surface inhomogeneity was reduced to one in the case of the l-alanine capped ZnO/CuI diode. Due to the exponent values near unity, even Qy1 had been taken as capacitance for the l-Alanine capped device. Thus, (i) the reduced number of CPEs, (ii) absence of void/CuI interface resistance and Warburg resistance in the equivalent circuit fitted for the Nyquist plot of the ZnO–l-Alanine/CuI diode in comparison with those of the ZnO/CuI diode demonstrated the surface passivation of the defects on ZnO by l-Alanine. Thus, EIS studies concluded that l-Alanine capping contributed to a smoother surface with increased homogeneity and thus, it avoided recombination of electrons and holes in the bulk. This homogeneity of the surface due to l-Alanine capping could be attributed to the strong and stabilized O–Zn–O=C interaction, as discussed in Section 3.5, by quantum chemical studies.

### 3.8 Heterojunction device characteristics

**Fig. 11:**
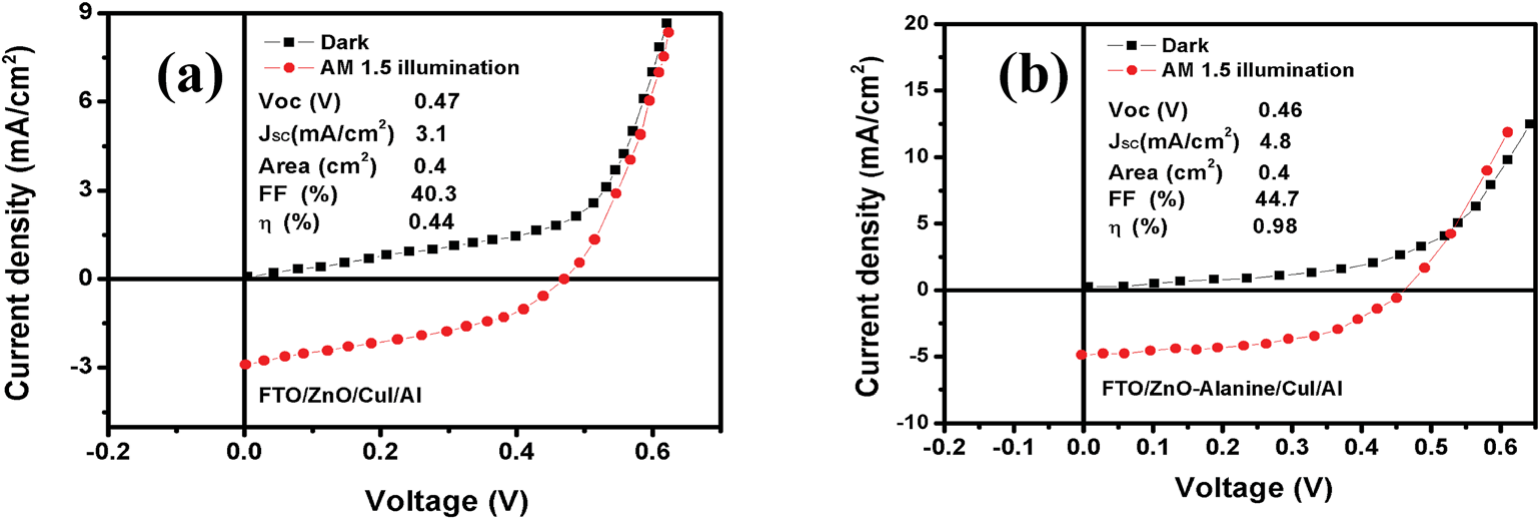
Current–voltage characteristics of (a) ZnO/CuI and (b) ZnO–l-Alanine/CuI heterojunction devices in the dark and at AM 1.5 illumination.

*I*–*V* characteristics of FTO/ZnO/CuI/Al heterojunction devices under the dark and illumination conditions are shown in Fig. 11. *I*–*V* characteristics were studied using the thermionic emission equation.^65^1
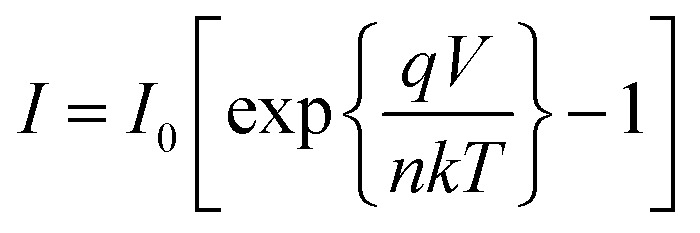
*I*_0_ is the saturation current and is written as^54^2
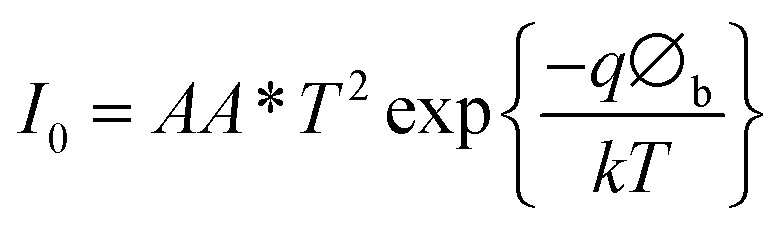
where *A* is the diode area, A* represents the Richardson constant (32 A cm^2^ K^−2^ for ZnO),^64^*k* depicts Boltzmann constant, *q* indicates the electronic charge, *η* denotes the ideality factor and *Φ*_b_ corresponds to the barrier height. From the linear fit to the semi-log plot of *I*–*V* (ESI†), the ideality factor and barrier height were calculated using the slope and the intercept. The ideality factor of 1.19 and 1.16 for ZnO/CuI and ZnO–l-Alanine/CuI heterojunction indicated that the recombination type was mostly band to band and it was limited by the minority carrier concentration.^46^ The diode characteristics of the heterojunction such as series resistance (*R*_s_) and shunt resistance (*R*_sh_) were calculated and are shown in Table 2. The devices under the dark were rectifying devices and exhibited excellent photovoltaic effect under AM 1.5 illumination. An open-circuit voltage (*V*_oc_) of 0.47 V and a short-circuit current density (*J*_sc_) of 3.1 mA cm^−2^ were observed for the ZnO/CuI heterojunction, while for ZnO–l-Alanine/CuI, *V*_oc_ was 0.46 V and *J*_sc_ was 4.8 mA cm^−2^ (Table 2). *J*_sc_ of the ZnO–l-Alanine/CuI heterojunction was 1.55 times higher than that of the ZnO/CuI heterojunction, while *V*_oc_ showed no significant change. The photovoltaic conversion efficiency obtained for the ZnO/CuI and ZnO–l-Alanine/CuI heterojunctions was 0.44% and 0.98%, respectively, showing 2.23 times increase in the efficiency after l-Alanine capping. This could be attributed to the increased carrier concentration of l-Alanine capped ZnO NRs due to the electron releasing +I effect of l-Alanine and its coordination bond with ZnO as discussed in Section 3.5.

**Table 2:** Photovoltaic properties of the ZnO/CuI and ZnO–l–Alanine/CuI heterojunction devices

S. no.	Device structure	Top electrode area (cm^2^)	Ideality factor (*I*_B_)	Valence band offset (eV)	*R*_s_ (Ω cm^2)^	*R*_sh_ (Ω cm^2)^	*V*_oc_ (V)	*J*_sc_ (mA cm^−2^)	FF	Efficiency *η* (%)
1	SLG/FTO/ZnO/CuI/Al	0.4	1.19	2.32	1.32	146	0.44	2.5	40.3	0.44
2	SLG/FTO/ZnO–l-Alanine/CuI/Al	0.4	1.16	2.25	0.71	79	0.46	4.8	44.7	0.98

**Fig. 12:**
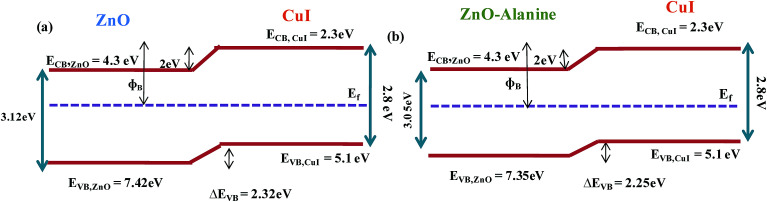
Energy level diagram of (a) ZnO/CuI, (b) ZnO–l-Alanine/CuI heterojunction devices.

The energy level diagram and energy band alignment of the ZnO–l-Alanine/CuI heterojunction are shown in Fig. 12. The Fermi level of the ZnO NRs and CuI were near the ZnO conduction band edge and CuI valence band edge, respectively. Considering the estimated band gap value obtained from our optical absorption studies for ZnO–l-Alanine (3.12 eV), the valence band offset in ZnO/CuI and ZnO–l-Alanine/CuI heterojunctions was calculated as 2.32 and 2.25 eV, respectively (Fig. 12). Thus, the effect of l-Alanine capping was also accompanied by a favourable shift in the energy levels to obtain more than two-fold increase in the efficiency.

## 4 Conclusions

ZnO NRs were capped with l-Alanine and the effect of capping on the properties of ZnO and ZnO/CuI heterojunction was studied. Quantum chemical studies demonstrated the existence of two equally strong Zn–O coordinate bonds with 70 kcal mol^−1^ stabilization energy in comparison with other structures considered. This stabilized structure was further confirmed by FTIR spectroscopy, showing the broadening of the high-intensity peak at 3398 cm^−1^ for O–Zn–O=COH coordination bonds. Hall effect experiments showed two orders of magnitude increase in the carrier concentration of ZnO NRs due to l-Alanine capping. This increase in carrier concentration for ZnO–l-Alanine could be attributed to the +I (inductive) effect of –CH_3_, –NH_2_ and –COOH groups of the alanine moiety. Electrochemical impedance spectroscopy studies concluded that the capping with ZnO–l-Alanine also contributed to a smoother surface and less recombination. The photovoltaic conversion efficiency of ZnO/CuI heterojunction was increased by 2.23 times due to the capping. Thus, this study introduces the use of +I effect *via* amino acid capping as a viable way to enhance the performance of nanomaterials such as ZnO in optoelectronic devices. Further, in addition to the inductive effect, the resonance effect due to conjugated or long-chain hydrocarbon moiety could be explored using amino acids such as phenylalanine or arginine.

## Authors contribution statement

EI carried out the electrochemical, structural and optical characterizations. SVB and MD carried out device fabrication, film coating, electrical property measurements and analysis. VS and SHP performed cyclic voltammetry experiments and analyzed data. EJP and VD carried out and analyzed the quantum mechanical calculations. JS synthesized ZnO and ZnO–l-Alanine and performed the dispersion experiments. RR synthesized CuI nanoparticles *via* a green route. SVB and SHP devised the idea and wrote the manuscript.

## Conflicts of interest

The authors declare no conflicts of interest.

## Supplementary Material

RA-008-C7RA12385J-s001

## References

[cit1] Saha S., Sarkar P. (2014). Controlling the electronic energy levels of ZnO quantum dots using mixed capping ligands. RSC Adv..

[cit2] Brown P. R., Kim D., Lunt R. R., Zhao N., Bawendi M. G., Grossman J. C., Vladimir B. (2014). Energy level modification in lead sulphide quantum dot thin films through ligand exchange. ACS Nano.

[cit3] Grandhi G. K., Arunkumar M., Viswanatha R. (2016). Understanding the role of surface capping ligands in passivating the quantum dots using copper dopants as internal sensors. J. Phys. Chem. C.

[cit4] Green M. (2010). The nature of quantum dot capping ligands. J. Mater. Chem..

[cit5] Ren Z., Yu J., Pan Z., Wang J., Zhong X. (2017). Inorganic ligand thisulphate – capped quantum dots for efficient quantum dot sensitized solar cells. ACS Appl. Mater. Interfaces.

[cit6] Thomas G. (1997). Materials science: Invisible circuits. Nature.

[cit7] Akasaki I., Amano H. (2006). Breakthroughs in improving crystal quality of GaN and invention of the p–n junction blue-light-emitting diode. Jpn. J. Appl. Phys..

[cit8] Grundmann M., Frenzel H., Lajn A., Lorenz M., Schein F., von Wenckstern H. (2010). Transparent semiconducting oxides: Materials and devices. Phys. Status Solidi A.

[cit9] Roy U. N., Mundle M., Camarda G. S., Cui Y., Gul R., Hossain A., Yang G., Pradhan A. K., James R. B. (2016). Novel ZnO: Al contacts to CdZnTe for X- and gamma-ray detectors. Sci. Rep..

[cit10] Konovalov L., Makhova M. L. (2007). Small valence band offset in (010) InS/CuI heterojunction diodes. Appl. Phys. Lett..

[cit11] Sun X. W., Kwok H. S. (1999). Optical properties of epitaxially grown zinc oxide films on sapphire by pulsed laser deposition. J. Appl. Phys..

[cit12] Talwatkar S. S., Sunatkari A. L., Tamgadge Y. S., Pahurkar V. G., Muley G. G. (2015). Surface passivation by l-Arginine and enhanced optical properties of CdS quantum dots co-doped with Nd^3+^–Li^+^. J. Nanostruct. Chem..

[cit13] Verbakel F., Meskers S. C. J., Janssen R. A. J. (2007). Surface Modification of Zinc Oxide Nanoparticles Influences the Electronic Memory Effects in ZnO−Polystyrene Diodes. J. Phys. Chem. C.

[cit14] Colbert A. E., Wu W., Janke E. M., Ma F., Ginger D. S. (2015). Effects of ligands on charge generation and recombination in hybrid polymer/quantum dot solar cells. J. Phys. Chem. C.

[cit15] Hewlett R. M., McLachlan M. A. (2016). Surface structure modification of ZnO and the impact on electronic properties. Adv. Mater..

[cit16] Bazargan A. M., Sharif F., Mazinani S., Naderi N. (2016). Integrated synthesis and surface passivation of ZnO nanoparticles to enhance UV spectrum selectivity. J. Mater. Sci.: Mater. Electron..

[cit17] Yang H., Wu T., Hu T., Hu X., Chen L., Chen Y. (2016). A homogeneous ethanedithiol doped ZnO electron transporting layer for polymer solar cells. J. Mater. Chem. C.

[cit18] Bollero A., Fernandez S., Rozman K. Z., Samardzija Z., Grossberg M. (2012). Preparation and quality assessment of CuS thin films encapsulated in glass. Thin Solid Films.

[cit19] Park J. S., Lee J. M., Hwang S. K., Lee S. H., Lee H.-J., Lee B. R., Park H. I., Kim J.-S., Yoo S., Song M. H. A. (2012). ZnO/N doped carbon nanotube nanocomposite charge transport layer for high performance optoelectronics. J. Mater. Chem..

[cit20] Park H.-Y., Lim D., Kim K.-D., Jang S.-Y. (2013). Perfromance optimization of low temperature annealed solution processable – ZnO buffer layers for inverted polymer solar cells. J. Mater. Chem. A.

[cit21] Liu J., Lu Y., Liu J., Yang X., Yu X. (2010). Investigation of near infrared reflectance by tuning the shape of SnO_2_ nanoparticles. J. Alloys Compd..

[cit22] Chuang C. H. M., Brown P. R., Bulović V., Bawendi M. G. (2014). Improved performance and stability in quantum dot solar cells through band alignment engineering. Nat. Mater..

[cit23] Tamgadge Y., Pahurkar V., Sunatkari A., Talwatkar S., Muley G. (2016). Thermo-Optical Properties of Amino Acid Modified ZnO-PVA Colloidal Suspension Under CW Laser Illumination. Macromol. Symp..

[cit24] Declan B. (2008). Thin films: Ready for their close-up. Nature.

[cit25] Nelwamondo S. M. M., Moloto M. J., Krause R. W. M., Moloto N. (2012). Synthesis and characterization of alanine-capped water soluble copper sulphide quantum dots. Mater. Lett..

[cit26] Cullity B. D. , Elements of X-Ray Diffraction, Addison-Wesley Publishing Company, Inc, Reading, Massachusetts, 2nd edn, 1956

[cit27] Jaeger H. M., Schaefer III H. F., Demaison J., Csaszar A. G., Allen W. D. (2010). Lowest-lying conformers of alanine: Pushing theory to ascertain precise energetics and semiexperimental *R*_e_ structures. J. Chem. Theory Comput..

[cit28] Zhao Y., Schultz N. E., Truhlar D. G. (2006). Design of density functionals by combining the method of constraint satisfaction with parametrization for thermochemistry, thermochemical kinetics, and noncovalent interactions. J. Chem. Theory Comput..

[cit29] Grimme S. (2006). Semiempirical hybrid density functional with perturbative second-order correlation. J. Chem. Phys..

[cit30] Schaefer A., Huber C., Ahlrichs R. (1994). Fully optimized contracted Gaussian basis sets of triple zeta valence quality for atoms Li to Kr. J. Chem. Phys..

[cit31] Weigend F., Ahlrichs R. (2005). Balanced basis sets of split valence, triple zeta valence and quadruple zeta valence quality for H to Rn: Design and assessment of accuracy. Phys. Chem. Chem. Phys..

[cit32] Weigend F. (2006). Accurate Coulomb-fitting basis sets for H to Rn. Phys. Chem. Chem. Phys..

[cit33] Yu F., Fu L.-X., Yang Y. (2017). DSD-PBEP86-NL and DOD-PBEP86-NL functionals for noncovalent interactions: Basis set effects and tentative applications to large noncovalent systems. Int. J. Quantum Chem..

[cit34] Bartlett R. J., Purvis G. D. (1978). Many-body perturbation theory, coupled-pair many-electron theory, and the importance of quadruple excitations for the correlation problem. Int. J. Quantum Chem..

[cit35] Lee T. J., Rendell A. P., Taylor P. R. (1990). Comparison of the Quadratic Configuration Interaction and Coupled-Cluster Electron Correlation Methods, Including the Effects of Triple Excitations. J. Phys. Chem..

[cit36] Liakos D. G., Neese F. (2015). Is it possible to obtain coupled cluster quality energies at near density functional theory cost? Domain-based local pair natural orbital coupled cluster *vs.* modern density functional theory. J. Chem. Theory Comput..

[cit37] Hellweg A., Hattig C., Hofener S., Klopper W. (2007). Optimized accurate auxiliary basis sets for RI-MP2 and RI-CC2 calculations for the atoms Rb to Rn. Theor. Chem. Acc..

[cit38] Frisch M. J. , et al., GAUSSIAN 03, Revision E.01, Gaussian, Inc., Wallingford CT, 2004

[cit39] Neese F. (2012). The ORCA program system. Wiley Interdiscip. Rev.: Comput. Mol. Sci..

[cit40] Izsak R., Neese F. (2011). An overlap fitted chain of spheres exchange method. J. Chem. Phys..

[cit41] Neese F. (2003). An improvement of the resolution of the identity approximation for the formation of the coulomb matrix. J. Comput. Chem..

[cit42] Kossmann S., Neese F. (2010). Efficient structure optimization with second-order many-body perturbation theory, The RIJCOSX-MP2 method. J. Chem. Theory Comput..

[cit43] Kossmann S., Neese F. (2009). Comparison of two efficient approximate Hartree-Fock approaches. Chem. Phys. Lett..

[cit44] Neese F., Wennmohs F., Hansen A., Becker U. A. (2009). ‘chain-of-spheres’ algorithm for the Hartree-Fock exchange. Chem. Phys..

[cit45] Kamarulzaman N., Kasim M. F., Rusdi R. (2015). Band Gap Narrowing and Widening of ZnO Nanostructures and Doped Materials. Nanoscale Res. Lett..

[cit46] Schein F. L., von Wenckstern H., Grundmann M. (2013). Transparent p-CuI/n-ZnO heterojunction diodes. Appl. Phys. Lett..

[cit47] Ghosh M., Raychaudhari A. K. (2006). Shape transition in ZnO nanostructures and its effects on blue-green photoluminescence. J. Appl. Phys..

[cit48] Shalish I., Temkin H., Narayananmurti V. (2004). Size dependent surface luminescence in ZnO nanowires. Phys. Rev..

[cit49] Mayer I. (1983). Charge, bond order and valence in the *ab initio* SCF theory. Chem. Phys. Lett..

[cit50] Mayer I. (1984). Bond order and valence: Relations to Mulliken's population analysis. Int. J. Quantum Chem..

[cit51] Mayer I. (1985). Bond orders and valences in the SCF theory: A comment. Theor. Chim. Acta.

[cit52] Malar E. J. P. (2005). Density functional theory analysis of some triple-decker sandwich complexes of iron containing cyclo-P5 and cyclo-As5 ligands. Theor. Chem. Acc..

[cit53] Malar E. J. P. (2003). Do penta- and decaphospha analogues of lithocene anion and beryllocene exist? Analysis of stability, structure, and bonding by hybrid density functional study. Inorg. Chem..

[cit54] Malar E. J. P. (2004). Can the cyclo-P5 ligand introduce basicity at the transition metal center in metallocenes? A hybrid density functional study on the cyclo-P5 analogues of metallocenes of Fe, Ru and Os. Eur. J. Inorg. Chem..

[cit55] Sankaran A., Malar E. J. P., Vijayaraghavan V. R. (2015). Kinetic measurements and quantum chemical calculations on low spin Ni(ii)/(iii) macrocyclic complexes in aqueous and sulphatomedium. J. Chem. Sci..

[cit56] Sankaran A., Malar E. J. P., Vijayaraghavan V. R. (2017). Study of behaviour of Ni(iii) macrocyclic complexes in acidic aqueous medium through kinetic measurement involving hydrogen peroxide oxidation and DFT calculations. J. Chem. Sci..

[cit57] Wendler K., Thar J., Zahn S., Kirchner B. (2010). Estimating the Hydrogen Bond Energy. J. Phys. Chem. A.

[cit58] Grimme S. (2011). Density functional theory with London dispersion corrections. Wiley Interdiscip. Rev.: Comput. Mol. Sci..

[cit59] Grimme S., Antony J., Ehrlich S., Krieg H. (2010). A consistent and accurate *ab initio* parametrization of density functional dispersion correction (DFT-D) for the 94 elements H-Pu. J. Chem. Phys..

[cit60] Goerigkab L., Grimme S. (2011). A thorough benchmark of density functional methods for general main group thermochemistry, kinetics, and noncovalent interactions. Phys. Chem. Chem. Phys..

[cit61] Zhao Y., Truhlar D. G. (2008). The M06 suite of density functionals for main group thermochemistry, thermochemical kinetics, noncovalent interactions, excited states, and transition elements: two new functionals and systematic testing of four M06-class functionals and 12 other functionals. Theor. Chem. Acc..

[cit62] Lambert J. F. (2008). Adsorption and polymerization of amino acids on mineral surfaces: A review. Origins Life Evol. Biospheres.

[cit63] Morrison R. T. and Boyd R. N., Organic Chemistry, Pearson Publications, 6th edn, 1992

[cit64] Sidhu N. K., Rastogi A. C. (2014). Vertically aligned ZnO nanorod core-polypyrrole conducting polymer sheath and nanotube arrays for electrochemical supercapacitor energy storage. Nanoscale Res. Lett..

[cit65] Charles K. , Introduction to Solid State Physics, John Wiley & Sons, 7th edn, 2004

