# Supplementary material for: l-Alanine capping of ZnO nanorods: increased carrier concentration in ZnO/CuI heterojunction diode
Source: RSC Adv. 2018 Jan 31;8(10):5350–61. doi: 10.1039/c7ra12385j (PMC9078177; doi:10.1039/c7ra12385j)
Supplement: RA-008-C7RA12385J-s001 [file RA-008-C7RA12385J-s001.pdf]

Electronic Supplementary Material (ESI) for RSC Advances.
This journal is © The Royal Society of Chemistry 2018

Electronic Supplementary Information

L – Alanine capping of ZnO nanorods: Increased carrier concentration in ZnO/CuI heterojunction diode

E. Indubala[a,c], M. Dhanasekar[b,e], V. Sudha[c], E. J. Padma Malar[d], P. Divya[d], Jositta Sherine[e], Revathy Rajagopal[f], S.Venkataprasad Bhat[b,e], S. Harinipriya[a,c*]

[a]*Electrochemical Systems Lab, SRM Research Institute, SRM Institute of Science and Technology, Kattankulathur, Chennai – 603203, India*

[b]*SRM Research Institute, SRM Institute of Science and Technlogy, Kattankulathur, Chennai – 603203, India*

[c]*Department of Chemistry, SRM Institute of Science and Technology, Kattankulathur, Chennai – 603203, India*

[d]*National Centre for Ultrafast Processes, University of Madras, Taramani Campus, Chennai - 600 113, India*

[e]*Department of Physics and Nanotechnology, SRM Institute of Science and Technology, Kattankulathur, Chennai – 603203, India*

[f]*Department of Chemistry, Stella Maris College, Chennai – 600086, India*

[a*]*Corresponding Author: Electrochemical Systems Lab, SRM Research Institute, SRM Institute of Science and Technology, Kattankulathur, Chennai – 603203, India*

Table of contents

1. Figure S1. XRD pattern of CuI (Syzygium Cumini)

2. Table S1: The peak position and corresponding crystallite size for CuI nanoparticles synthesized from syzygium cumini extract.
3. Figure S2 FESEM and EDS of CuI nanoflowers extracted from Syzygium cumini plant extract
4. Figure S3 (a) Fabrication process of ZnO-CuI heterojunction, (b) Photographs of heterojunction device fabricated, (c) Photographs of ZnO NRs, ZnO-L-Alanine NRs and heterojunction devices on FTO coated glass.
5. Table S2. Total energies of alanine-ZnO at B2PLYP/def2-TZVP, M052X/TZVP and single-point CCSD(T)/TZVP levels

6. Table S3. Total energy of alanine-ZnO at single-point DLPNO-CCSD(T)/def2-TZVP and CCSD(T)/QZVP//M052X/TZVP levels.

7. Table S4. Cartesian coordinates in Å of the B2PLYP/def2-TZVP optimized geometries of alanine-ZnO.

8. Table S5. Cartesian coordinates in Å of the M052X/TZVP optimized geometries of alanine-ZnO.

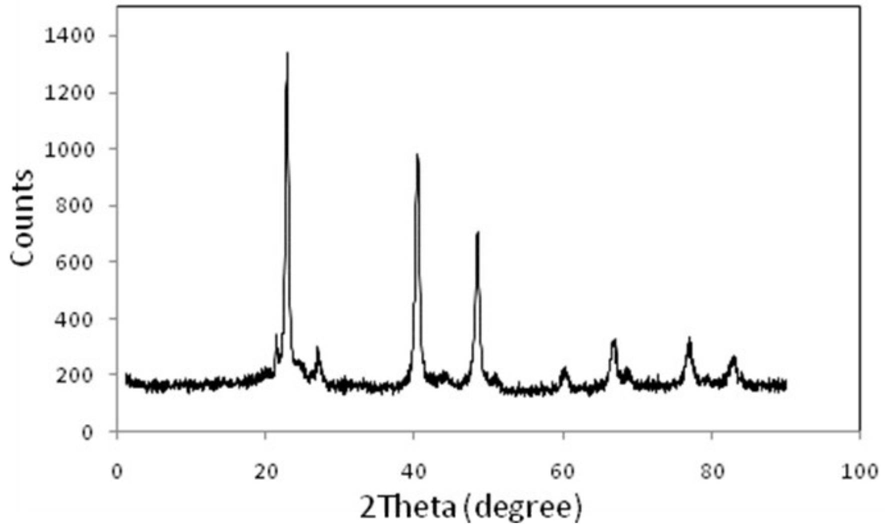

Figure S1 XRD pattern of CuI (Syzygium Cumini)

The powder X-ray patterns of the CuI nanoparticles synthesized from Syzygium cumini seed agrees well with JCPDS.No.(82–2111). CuI is found to crystallize in cubic phase with a space group of *F- 43m*. No significant impurities were observed in the XRD patterns, indicating high purity of the products. All the reflections correspond to pure CuI particles with face centered cubic symmetry. The high intense peak for *fcc* materials is generally (111) reflection, which is observed in the sample. The intensity of the peaks reflected high degree of crystallinity of the synthesized products. However, the diffraction peaks are broad which may be attributed to the small crystallite size. Using Scherer formula the average particle size of CuI particles were found to be ~ 28 *nm*. Table S1 shows the peak position and corresponding crystallite size for CuI nanoparticles synthesized from Syzygium cumini.

Table S1: The peak position and corresponding crystallite size for CuI nanoparticles synthesized from syzygium cumini extract.

S.No	Peak Position	Crystallite size(nm)
1.	21.338	44.20
2.	22.844	31.90
3.	27.064	17.5
4.	40.388	57.2
5.	48.488	47.6
6.	60.215	12.2
7.	66.786	17.7
8.	76.953	13.5
9.	82.966	11.3

FESEM analysis

The FESEM images of CuI nanoparticles synthesized using Syzygium cumini seeds appear to be spheres made of flakes. The particles seem to have flower like structures. The mean particle size of CuI from syzygium cumini seed extract is found to be 28.12 *nm*. EDS of synthesized nanoparticles show that they are made of pure CuI.

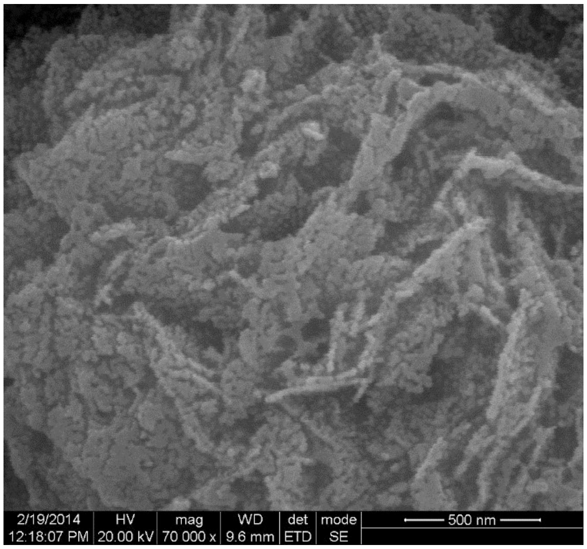

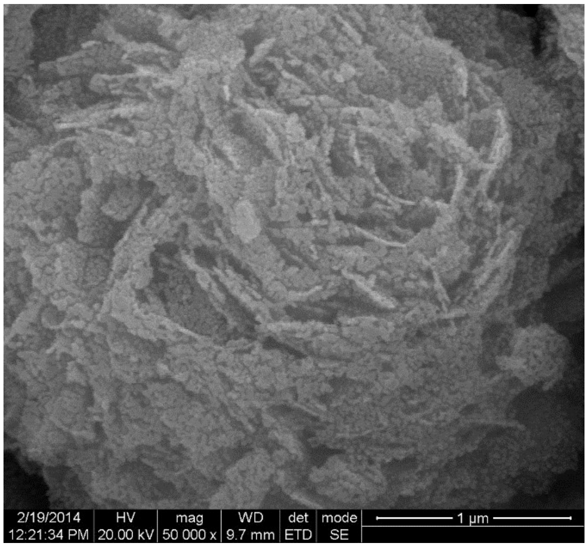

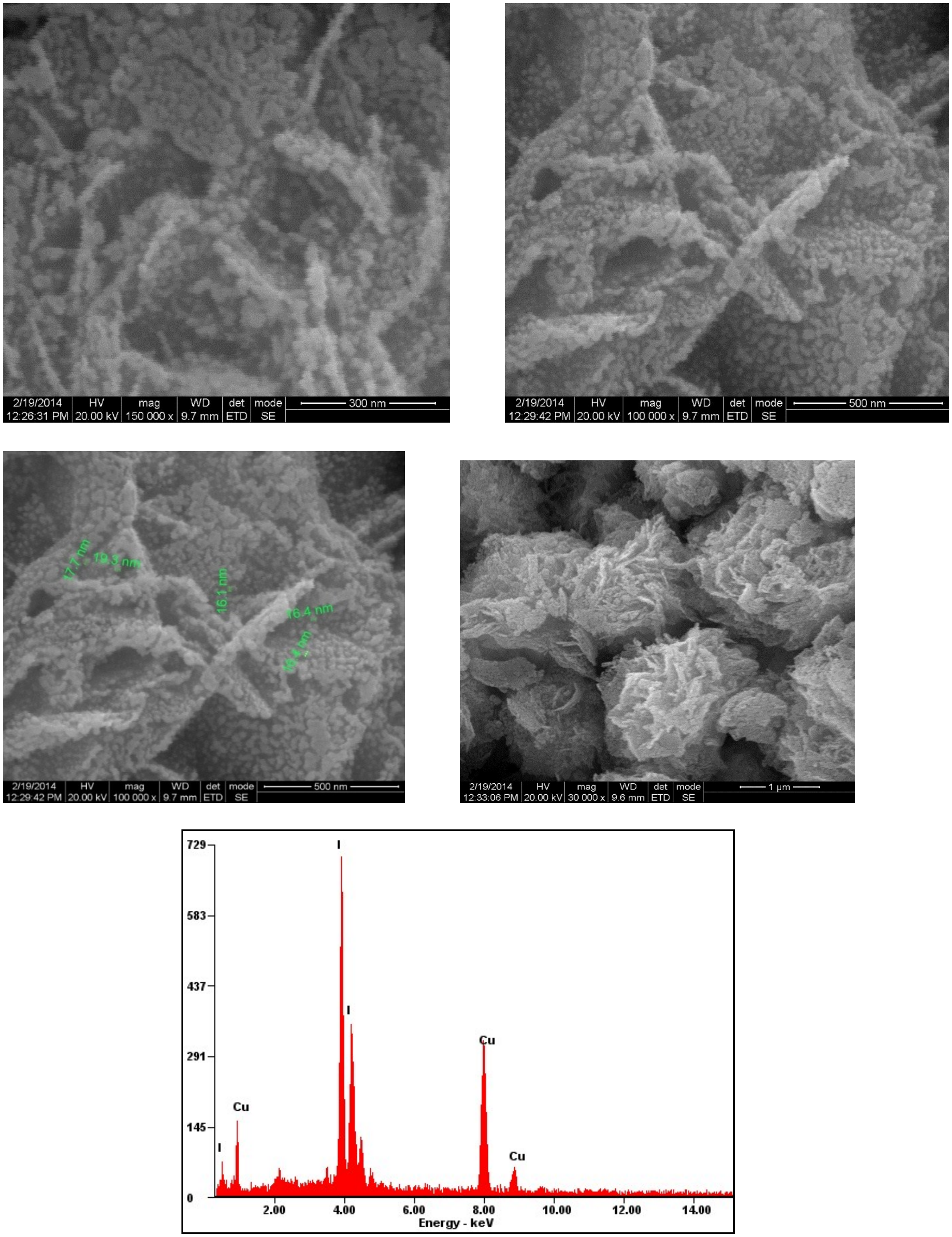

Figure S2 FESEM and EDS of CuI nanoflowers extracted from Syzygium cumini plant extract

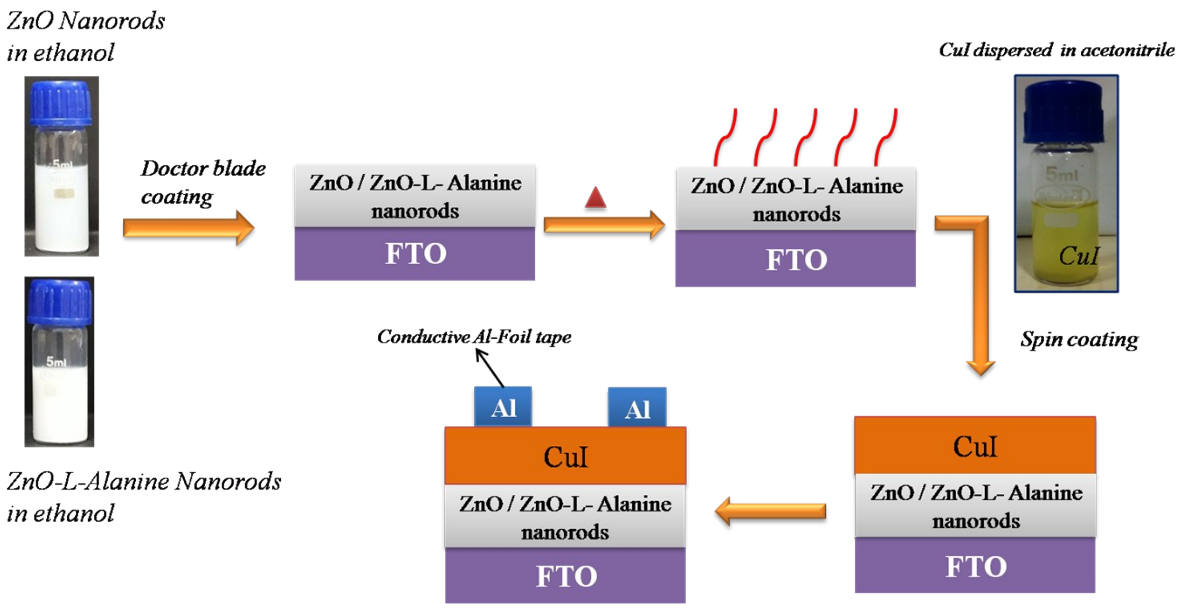

Figure S3 (a) Fabrication process of ZnO-CuI heterojunction

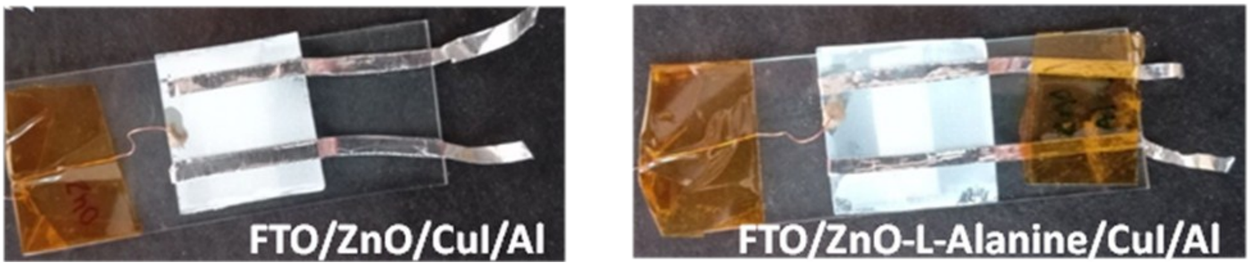

Figure S3 (b) Photographs of heterojunction device fabricated

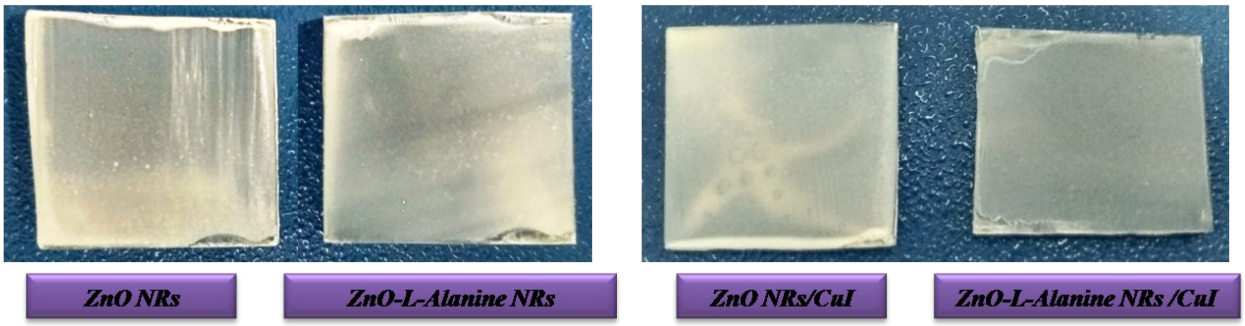

Figure S3 (c) Photographs of ZnO NRs, ZnO-L-Alanine NRs and heterojunction devices on FTO coated glass.

Table S2. Total energy (hartree) of alanine-ZnO at B2PLYP/def2-TZVP, M052X/TZVP and single point CCSD(T)/TZVP levels. Total energy (TE) and Zero-point vibrational energy (ZPE) in hartree and relative energy (RE) in kcal/mol.

Structure	B2PLYP /def2-TZVP	M052X/TZVP	M052X/TZVP	CCSD(T)/TZVP//B2PLYP/def2-TZVP			CCSD(T)/TZVP//M052X/TZVP		
	TE	TE	ZPE	TE	TE+ZPE	RE	TE	TE+ZPE	RE
**A**	-2177.994063	-2178.306207	0.113131	-2176.329579	-2176.216447	82.10	-2176.329617	-2176.216485	81.73
**B**	-2178.109195	-2178.440831	0.112131	-2176.459407	-2176.347276	0.0	-2176.458859	-2176.346728	0.0
**C**	-2178.00552	-2178.310329	0.114778	-2176.337555	-2176.222777	78.12	-2176.338235	-2176.223457	77.35
**D**	-2178.007966	-2178.311342	0.114845	-2176.338653	-2176.223808	77.48	-2176.339367	-2176.224522	76.69
**E**	-2177.984162	-2178.285474	0.113298	-2176.313285	-2176.199987	92.43	-2176.312944	-2176.199646	92.30
**F**	-2177.969984	-2178.271461	0.112089	-2176.302859	-2176.19077	98.21	-2176.302171	-2176.190082	98.30

Table S3. Total energy (hartree) of alanine-Zno at single point DLPNO-CCSD(T)/def2-TZVP and CCSD(T)/QZVP//M052X/TZVP levels. Zero-point vibrational energy (ZPE in hartree) given in Table S1 is used to get relative energy (RE in kcal/mol). Dispersion energy (kcal/mol) is obtained by M062X-D3/def2-TZVP//M052X/TZVP calculation.

Structure	DLPNO-CCSD(T)/def2-TZVP//B2PLYP/def2-TZVP			DLPNO-CCSD(T)/def2-TZVP//M052X/TZVP			CCSD(T)/QZVP//M052X/TZVP			Dispersion energy
	TE	TE+ZPE	RE	TE	TE+ZPE	RE	TE	TE+ZPE	RE	
**A**	-2176.708085	-2176.594953	79.40	-2176.707174	-2176.59404	79.59	-2177.087329	-2176.974198	76.90	-0.25
**B**	-2176.833622	-2176.721491	0.0	-2176.833009	-2176.72088	0.0	-2177.208879	-2177.096748	0.0	-0.26
**C**	-2176.719733	-2176.604955	73.13	-2176.717803	-2176.60303	73.95				-0.27
**D**	-2176.721718	-2176.606873	71.92	-2176.720459	-2176.60561	72.33				-0.26
**E**	-2176.696964	-2176.583666	86.49	-2176.696529	-2176.58323	86.37				-0.28
**F**	-2176.683410	-2176.571321	94.23	-2176.682932	-2176.57084	94.15				-0.27

Table S4. Cartesian coordinates in Å of the B2PLYP/def2-TZVP optimized geometries

a) Structure **A** **total energy** = -2177.994063 h

Zn	-1.139207	-0.209787	-1.021391
O	-1.464686	-1.700278	-1.771379
C	1.669040	-0.416633	0.567328
C	0.929460	0.917522	0.555611
O	1.583454	1.890671	1.163371
H	1.074278	2.715008	1.076563
O	-0.166845	1.153915	0.036782
N	0.851842	-1.588984	0.707460
C	2.491532	-0.497763	-0.724197
H	3.102438	-1.396467	-0.679096
H	1.833695	-0.577076	-1.590426
H	3.139983	0.369159	-0.841961
H	2.354439	-0.351494	1.414416
H	0.332750	-1.873680	-0.124045
H	0.248048	-1.589969	1.516653

b) Structure **B** total energy =-2178.109195h

O	-3.308373	0.081925	0.277987
Zn	-1.540962	-0.026836	0.027967
C	0.805071	-0.033744	-0.216822
O	0.098768	-0.963555	-0.724430
O	0.223398	0.899573	0.421279
H	-3.808741	-0.633346	-0.126019
C	2.316532	-0.067119	-0.355150
H	2.520778	-0.384720	-1.379163
N	2.982968	1.200970	-0.124202

C	2.865950	-1.130365	0.597804
H	2.404353	-2.098112	0.412045
H	3.941714	-1.209839	0.457804
H	2.674816	-0.846260	1.634007
H	2.650975	1.902675	-0.774739
H	2.756351	1.544096	0.802701

c) Structure **C** total energy =-2178.00552 h

Zn	-1.079575	0.976460	1.097361
O	-1.875375	0.720147	2.563541
C	1.416077	0.795295	-0.525440
C	1.350383	-0.688113	-0.851969
O	2.471510	-1.333418	-0.515189
H	2.365416	-2.261629	-0.780629
O	0.407970	-1.203205	-1.399509
N	0.017525	1.300739	-0.574782
C	2.092870	1.124137	0.795973
H	2.072597	2.199743	0.969009
H	1.588929	0.634318	1.629432
H	3.129642	0.798396	0.775519
H	1.986886	1.244999	-1.344699
H	0.019713	2.297639	-0.770575
H	-0.459827	0.833560	-1.345746

d) Structure **D** total energy =-2178.007966 h

O	-3.475963	0.088044	-0.674269
Zn	-1.903678	-0.355550	-0.256776
N	0.019099	-0.757141	0.183504
H	0.313334	-1.613577	-0.283875

C	0.955332	0.333144	-0.180259
C	2.377340	-0.202360	-0.183109
O	3.257425	0.736281	-0.555981
H	4.139530	0.330594	-0.556491
H	0.121338	-0.959393	1.176581
O	2.673937	-1.336171	0.099686
C	0.783980	1.522605	0.755943
H	1.447212	2.330323	0.458921
H	-0.242908	1.883671	0.718228
H	1.019941	1.243463	1.784238
H	0.723412	0.641046	-1.201665

e) Structure **E** total energy =-2177.9841626h

O	3.491774	0.087203	0.281984
Zn	1.821160	-0.148816	0.181723
C	-0.996988	0.326142	-0.328953
O	-0.620923	1.510106	-0.785790
O	-0.143472	-0.454164	0.075670
C	-2.479966	-0.011263	-0.278739
H	-2.954685	0.470212	-1.139473
N	-2.770871	-1.421960	-0.335822
C	-3.052911	0.593721	1.008384
H	-2.882489	1.668082	1.070303
H	-4.122808	0.402501	1.035900
H	-2.591796	0.124966	1.878040
H	-2.410383	-1.848492	-1.180266
H	-2.357525	-1.908246	0.451103

H	-1.375371	2.031198	-1.095929

f) Structure **F** total energy -2177.9699841h

O	3.675458	0.128281	0.192116
Zn	2.004055	0.193887	-0.055285
C	-1.029717	-0.356584	-0.020705
O	0.004833	0.488271	-0.470307
O	-0.718007	-1.323055	0.592997
C	-2.434726	0.119349	-0.370242
H	-2.416042	0.473572	-1.405765
N	-3.426155	-0.923024	-0.271753
C	-2.804240	1.286720	0.550475
H	-2.101130	2.115941	0.476586
H	-3.795809	1.642118	0.281316
H	-2.824355	0.953019	1.588201
H	-3.210911	-1.701059	-0.882849
H	-3.468747	-1.287939	0.672820
H	-0.306501	1.245742	-0.986316

Table S5. Cartesian coordinates in Å of the M052X/TZVP optimized geometries

a) Structure **A** total energy =-2178.306207 h

Zn	1.330776	-0.563278	-0.065712
O	2.330349	0.831736	-0.145087
C	-1.336305	0.966868	0.291396
C	-1.433816	-0.544586	0.107030
O	-2.659822	-1.001247	0.003583
H	-2.635889	-1.955843	-0.163733
O	-0.488221	-1.336086	0.025542
N	-0.293323	1.414567	1.153785
C	-1.139774	1.567104	-1.108964
H	-1.246112	2.644496	-1.021772
H	-0.127762	1.358279	-1.457808
H	-1.873019	1.187921	-1.818069

H	-2.312923	1.265097	0.672597
H	0.655615	1.491513	0.763430
H	-0.329015	1.113376	2.111129

b) Structure **B** total energy =-2178.440831 h

O	-3.308823	0.125334	0.234066
Zn	-1.532671	0.007969	0.006105
C	0.813768	-0.028744	-0.229131
O	0.105330	-0.996696	-0.642140
O	0.232112	0.958893	0.308296
H	-3.865348	-0.588263	-0.075651
C	2.321976	-0.082090	-0.352326
H	2.534205	-0.424275	-1.364619
N	2.989984	1.180954	-0.127799
C	2.833929	-1.133066	0.633029
H	2.363003	-2.095292	0.452284
H	3.910639	-1.221242	0.519526
H	2.618644	-0.821422	1.655282
H	2.671733	1.880503	-0.782800
H	2.770394	1.527400	0.796200

c) Structure **C** total energy =-2178.310329 h

Zn	1.433265	-0.119929	0.159083
O	2.348893	-0.526771	-1.212554
C	-1.215460	0.869195	0.393848
C	-1.708145	-0.527368	0.068015
O	-2.605477	-0.531570	-0.912951
H	-2.871282	-1.447659	-1.076666

O	-1.348653	-1.507144	0.660777
N	-0.051564	0.694337	1.298671
C	-0.837383	1.671273	-0.845285
H	-0.440605	2.640901	-0.549002
H	-0.083084	1.149072	-1.437710
H	-1.719734	1.828454	-1.458022
H	-2.029790	1.367297	0.924289
H	0.211733	1.585613	1.700737
H	-0.296424	0.059122	2.051558

d) Structure **D** total energy =-2178.311342 h

O	-3.243340	0.652502	-0.139258
Zn	-1.824257	-0.267337	-0.048117
C	0.976363	0.256407	-0.253341
C	2.422719	-0.162611	-0.089740
O	3.265197	0.765467	-0.545289
O	2.767712	-1.199067	0.406701
H	4.170202	0.457552	-0.396046
N	0.101439	-0.894547	0.064678
C	0.656364	1.444355	0.650842
H	-0.372612	1.770892	0.495273
H	0.785819	1.169478	1.697593
H	1.323942	2.269486	0.421426
H	0.315319	-1.670189	-0.553856
H	0.337241	-1.229562	0.995233
H	0.808508	0.534159	-1.292653

e) Structure **E** total energy =-2178.285474 h

O	3.504626	0.128751	0.144763
Zn	1.837632	-0.173523	0.079174
C	-0.982445	0.292708	-0.292444
O	-0.603101	1.519955	-0.589456
O	-0.131494	-0.530598	0.001702
C	-2.466068	-0.033976	-0.293129
H	-2.893285	0.419544	-1.190811
N	-2.757022	-1.439510	-0.302858
C	-3.089920	0.625521	0.942353
H	-2.944268	1.703766	0.960114
H	-4.154168	0.411537	0.937926
H	-2.652252	0.205941	1.846747
H	-2.371281	-1.907423	-1.109306
H	-2.399867	-1.894494	0.525248
H	-1.344338	2.092994	-0.821890

f) Structure **F** total energy =-2178.271461 h

O	3.595730	-0.102968	0.184152
Zn	1.918564	0.057634	-0.029015
C	-1.054415	-0.306215	-0.066075
O	-0.040125	0.583594	-0.442801
O	-0.714632	-1.308318	0.460835
C	-2.468286	0.162513	-0.367708
H	-2.486022	0.502000	-1.406047
N	-3.443879	-0.880644	-0.207196
C	-2.801082	1.340670	0.552230
H	-2.126191	2.184084	0.419479
H	-3.814392	1.664249	0.335505
H	-2.749907	1.024775	1.593067

H	-3.257023	-1.670952	-0.806792
H	-3.462886	-1.215390	0.746163
H	-0.338427	1.386458	-0.888737
